# Comparative Performance of HALP, PNI, and CONUT Scores in No-Reflow Among Patients with Acute Coronary Syndrome: A Prospective Study

**DOI:** 10.3390/jcm15135191

**Published:** 2026-07-02

**Authors:** Mert Deniz Savcilioglu, Nil Savcilioglu, Nezihe Otay Lule, Osman Büyükcelebi, Mehmet Murat Sucu

**Affiliations:** 1 Cardiology Department, Faculty of Medicine, Gaziantep University, 27310 Gaziantep, Turkey; ncelikkalkan@gmail.com (N.S.); dr.buyukcelebi02@gmail.com (O.B.); mmuratsucu@gmail.com (M.M.S.); 2 Department of Nutrition and Dietetics, Faculty of Health Sciences, Gaziantep University, 27310 Gaziantep, Turkey; otaynezihe@hotmail.com

**Keywords:** no-reflow, CONUT score, PNI, HALP score, coronary microvascular dysfunction

## Abstract

**Background:** Nutritional impairment has been associated with adverse outcomes in acute coronary syndrome (ACS), yet its relationship with the no-reflow phenomenon remains incompletely understood. We aimed to compare the performance of the Hemoglobin–Albumin–Lymphocyte–Platelet (HALP), Prognostic Nutritional Index (PNI), and Controlling Nutritional Status (CONUT) scores for no-reflow assessment in patients with ACS. **Methods:** This prospective single-centre study included 279 consecutive patients with ACS undergoing percutaneous coronary intervention. HALP, PNI, and CONUT scores were calculated from admission laboratory parameters. No-reflow was defined as post-procedural TIMI flow grade ≤ 2 in the absence of mechanical obstruction, with myocardial blush grade used in equivocal cases. Hierarchical logistic regression, receiver operating characteristic (ROC) analysis, net reclassification improvement (NRI), integrated discrimination improvement (IDI), decision curve analysis, and bootstrap validation were performed. **Results:** No-reflow occurred in 46 patients (16.5%). All three nutritional indices were significantly associated with no-reflow (all *p* < 0.001). In multivariable analysis, only the CONUT score remained independently associated with no-reflow (OR 1.728, 95% CI 1.226–2.435, *p* = 0.002). The addition of nutritional indices to the clinical model improved discrimination, increasing the area under the curve from 0.649 to 0.693 for HALP, 0.733 for PNI, and 0.770 for CONUT. CONUT provided the largest likelihood-ratio improvement (χ^2^ = 25.98, *p* < 0.001), NRI (0.757, *p* < 0.001), and IDI (0.104, *p* < 0.001). Pairwise DeLong comparisons showed no statistically significant differences among the nutritional models. Internal validation of the CONUT model demonstrated good discrimination and calibration (bootstrap-corrected AUC 0.754). **Conclusions:** Among the evaluated nutritional indices, CONUT showed the largest incremental improvement in model performance; however, statistically significant superiority over HALP and PNI was not demonstrated. These findings should be considered as exploratory and require confirmation in larger multicentre studies.

## 1. Introduction

Percutaneous coronary intervention (PCI) remains the cornerstone of reperfusion therapy in acute coronary syndrome (ACS), restoring epicardial coronary patency in the large majority of patients. However, successful recanalisation of the culprit artery does not invariably translate into adequate myocardial tissue perfusion. The no-reflow phenomenon, defined as persistent impairment of microvascular perfusion in the absence of significant epicardial obstruction, complicates PCI in approximately 15–25% of cases and constitutes an independent predictor of larger infarct size, ventricular dysfunction, and both short- and long-term mortality [1,2,3].

The pathogenesis of no-reflow is multifactorial, encompassing distal embolisation, ischaemia–reperfusion injury, neutrophil and platelet activation, endothelial dysfunction, and microvascular spasm [4]. Systemic factors capable of amplifying this substrate, such as a pro-inflammatory milieu, oxidative stress, and impaired vascular homeostasis, are therefore of considerable mechanistic interest. Malnutrition has been associated with alterations in several of these pathways, including increased generation of reactive oxygen species, upregulation of pro-inflammatory cytokines, platelet hyperactivity, and impaired endothelial vasomotor reserve [5,6]. A direct relationship between malnutrition and coronary microvascular dysfunction has been documented, even in the absence of obstructive coronary artery disease, supporting biological plausibility for a nutritional contribution to no-reflow susceptibility [7].

Several composite nutritional indices derivable from routine admission laboratory values have been validated as prognostic tools in ACS. The Prognostic Nutritional Index (PNI), calculated from serum albumin and lymphocyte count, has been associated with long-term mortality and major adverse cardiovascular events (MACE) after PCI [8]. The Controlling Nutritional Status (CONUT) score, which incorporates albumin, total lymphocyte count, and total cholesterol, has similarly been linked to MACE and in-hospital mortality in patients with ACS undergoing revascularisation [9]. More recently, the Hemoglobin–Albumin–Lymphocyte–Platelet (HALP) score, a multidimensional index integrating nutritional, inflammatory, hematological, and thrombotic components, has emerged as an independent predictor of no-reflow and short-term mortality in patients with ST-elevation myocardial infarction (STEMI) undergoing primary PCI [10,11].

To our knowledge, no prospective study has directly compared HALP, PNI, and CONUT scores in a mixed ACS population with respect to no-reflow occurrence. Despite growing evidence linking nutritional status to adverse cardiovascular outcomes, the relative performance of these indices for no-reflow assessment and the degree to which they contribute incremental predictive information beyond conventional clinical variables remains unclear.

Therefore, this study had two principal aims. First, we sought to evaluate the association between HALP, PNI, and CONUT scores, calculated from routine admission laboratory parameters, and no-reflow in a prospective cohort of consecutive ACS patients undergoing PCI. Second, we aimed to compare the incremental discriminative and reclassification performance of each index beyond a clinical reference model, using a pre-specified analytical framework encompassing hierarchical logistic regression, receiver operating characteristic analysis, net reclassification improvement, integrated discrimination improvement, decision curve analysis, and bootstrap internal validation. By directly comparing all three indices within the same prospective cohort and across a consistent model-building hierarchy, we sought to provide a more complete and clinically interpretable characterisation of their relative utility for procedural risk stratification in ACS.

## 2. Materials and Methods

### 2.1. Study Design and Population

This was a single-centre prospective observational study conducted at the Department of Cardiology, Gaziantep University Sahinbey Research and Application Hospital, Gaziantep, Türkiye. The study was initiated following ethics committee approval (approval date: 4 February 2026; decision number: 2026/105) and consecutive patients with acute coronary syndrome (ACS) who underwent percutaneous coronary intervention (PCI) were enrolled between 5 February and 5 May 2026. Our institution serves as the primary tertiary cardiac referral centre for a catchment population of approximately 2.2 million, with 6437 coronary angiographic procedures performed in the preceding calendar year; enrolment of 279 consecutive eligible patients over the three-month study period is therefore consistent with routine institutional throughput.

Patients were eligible for inclusion if they fulfilled all of the following criteria: (1) age 18–70 years (an upper age limit of 70 years was applied to reduce the confounding effect of age-related comorbidities; including frailty, sarcopenia, and multimorbidity—on nutritional indices, given that these conditions independently alter albumin, lymphocyte count, and cholesterol levels irrespective of acute nutritional status); (2) a diagnosis of ACS confirmed by clinical presentation, electrocardiographic changes, and cardiac biomarker elevation with index-admission coronary angiography; (3) the availability of complete admission laboratory data required for nutritional score calculation; (4) coronary angiographic images of adequate quality and proper archival; and (5) the provision of written informed consent.

Patients were excluded if they had: (1) previous coronary artery disease requiring revascularisation (prior PCI or CABG); (2) significant left main coronary artery disease or culprit left main lesion; (3) active or recent malignancy; (4) active infection, systemic inflammatory or autoimmune disease; (5) advanced renal or hepatic failure; (6) chronic anti-inflammatory or immunosuppressive therapy; (7) pregnancy or lactation; (8) missing laboratory parameters required for nutritional score calculation; or (9) angiographic images unsuitable for evaluation.

**Figure 1:**
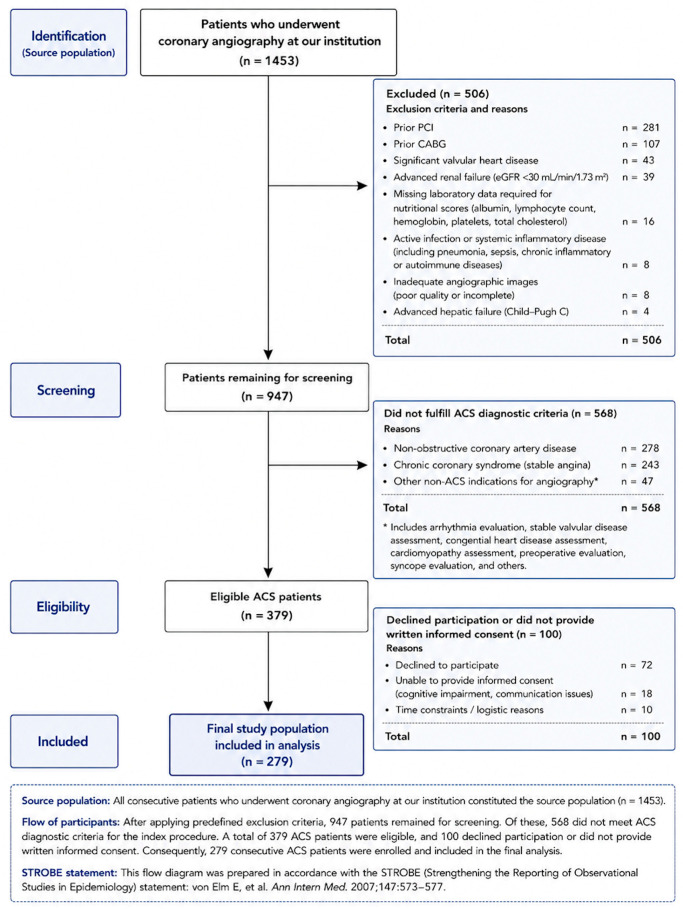
STROBE flow diagram illustrating patient selection and cohort derivation [12]. During the study period, 1453 patients underwent coronary angiography. After the application of predefined exclusion criteria, 947 patients remained for screening. Among these, 568 patients did not fulfil diagnostic criteria for acute coronary syndrome (ACS), including patients with non-obstructive coronary artery disease, chronic coronary syndrome, and other non-ACS indications for coronary angiography. A total of 379 patients fulfilled the eligibility criteria for ACS; 100 patients declined participation or did not provide written informed consent. Consequently, 279 consecutive patients constituted the final study cohort.

During the study period, 1453 patients underwent coronary angiography. After the exclusion of 506 patients according to predefined exclusion criteria, 947 patients remained for screening. Among these, 568 patients did not fulfil the diagnostic criteria for acute coronary syndrome (ACS), including patients with non-obstructive coronary artery disease (*n* = 278), chronic coronary syndrome (*n* = 243), and other non-ACS indications for coronary angiography such as heart failure evaluation, preoperative assessment, and arrhythmia investigation (*n* = 47). A total of 379 patients fulfilled the eligibility criteria for ACS. Of these, 100 patients declined participation or did not provide written informed consent. Consequently, 279 consecutive patients were enrolled and constituted the final study population (Figure 1).

### 2.2. Ethics Statement

The study protocol was approved by the Gaziantep University Non-Interventional Clinical Research Ethics Committee (approval date: 4 February 2026; decision number: 2026/105). The study was conducted in full accordance with the ethical principles of the Declaration of Helsinki and its subsequent amendments. Written informed consent was obtained from all participants prior to enrolment. Patients were explicitly informed that participation would have no influence on their routine clinical management and that withdrawal at any time would not affect their care.

### 2.3. Clinical and Laboratory Data Collection

Baseline demographic characteristics, cardiovascular risk factors, comorbidities, and clinical presentation data were collected prospectively from electronic medical records and standardised patient interviews at the time of enrolment. Clinical presentation was classified as STEMI or NSTEMI according to the 2023 ESC Guidelines for the management of acute coronary syndromes [1]. Information regarding previous coronary revascularisation procedures was verified through hospital records and patient interviews.

Venous blood samples were obtained at the time of initial hospital admission, prior to coronary angiography and before any invasive procedure or pharmacological intervention. Complete blood count parameters including hemoglobin, lymphocyte count, and platelet count were measured using an automated hematology analyser (Sysmex XN-1000, Sysmex Corporation, Kobe, Japan) employing impedance and flow cytometry techniques. Serum albumin and total cholesterol were quantified using an automated chemistry analyser (Roche Cobas 8000, Roche Diagnostics, Mannheim, Germany) with standardised enzymatic and immunoassay methods, in accordance with manufacturer protocols. All analyses were performed in the central laboratory of our institution.

### 2.4. Nutritional Score Calculations

Three established composite nutritional indices were calculated from admission laboratory values using the following standardised formulae.

The HALP (Hemoglobin–Albumin–Lymphocyte–Platelet) score was calculated as: HALP = [Hemoglobin (g/L) × Albumin (g/L) × Lymphocyte count (/L)]/Platelet count (/L).

The Prognostic Nutritional Index (PNI) was calculated as: PNI = 10 × Albumin (g/dL) + 0.005 × Total lymphocyte count (/mm^3^).

The Controlling Nutritional Status (CONUT) score was derived by assigning weighted points to three laboratory parameters: serum albumin (≥3.5 g/dL = 0 points; 3.0–3.49 = 2; 2.5–2.99 = 4; <2.5 = 6), total lymphocyte count (≥1600/mm^3^ = 0; 1200–1599 = 1; 800–1199 = 2; <800 = 3), and total cholesterol (≥180 mg/dL = 0; 140–179 = 1; 100–139 = 2; <100 = 3). The composite score ranges from 0 to 12, with higher scores indicating worse nutritional status (normal: 0–1; mild malnutrition: 2–4; moderate: 5–8; severe: 9–12).

### 2.5. Coronary Angiography and No-Reflow Assessment

Coronary angiography and PCI were performed using the standard Judkins technique with a Siemens Artis Zee floor-mounted digital flat-panel angiography system (Siemens Healthineers, Erlangen, Germany). All angiographic acquisitions were performed in multiple standard projections following intracoronary nitrate administration to exclude vasospasm and ensure optimal vessel visualisation.

No-reflow was defined as post-procedural TIMI flow grade ≤2 in the absence of mechanical obstruction (coronary dissection, spasm, or residual thrombus). In borderline or equivocal cases, myocardial blush grade (MBG) was additionally assessed; MBG 0–1 in the presence of TIMI grade 2 flow was considered indicative of impaired microvascular perfusion and supportive of the no-reflow diagnosis. TIMI flow was graded as: 0 (no perfusion), 1 (penetration without perfusion), 2 (partial perfusion), and 3 (complete perfusion). MBG was graded from 0 (absent blush) to 3 (normal blush) [5].

Pre-procedural TIMI Flow Grade (TFG) was assessed in all patients at the time of diagnostic angiography, prior to any intervention, as an objective index of baseline coronary perfusion status. TFG was graded as: 0 (no perfusion), 1 (penetration without perfusion), 2 (partial perfusion), and 3 (complete perfusion), consistent with the original TIMI classification [6].

All angiographic analyses were independently performed by two experienced interventional cardiologists blinded to clinical characteristics, laboratory data, and nutritional scores. Disagreements were resolved by a third senior interventional cardiologist, with a consensus decision applied in all cases.

### 2.6. Statistical Analysis

All statistical analyses were performed using R (version 4.3.2; R Foundation for Statistical Computing, Vienna, Austria).

Normality of continuous variables was assessed using the Shapiro–Wilk test supplemented by visual inspection of histograms and Q–Q plots. Normally distributed variables are presented as mean ± standard deviation (SD) and compared using the independent samples *t*-test. Non-normally distributed variables are presented as median [interquartile range (IQR)] and compared using the Mann–Whitney *U* test. Categorical variables are expressed as count (%) and compared using Pearson’s χ^2^ test or Fisher’s exact test, as appropriate. All tests were two-tailed; a *p* value < 0.05 was considered statistically significant.

Associations between nutritional indices and no-reflow were quantified using Spearman rank correlation analysis. Univariate binary logistic regression was performed to identify candidate predictors of no-reflow; results are reported as odds ratios (OR) with 95% confidence intervals (CI). Variables reaching *p* < 0.05 on univariate analysis were entered into the multivariable model using a forced-entry approach. Candidate variables for inclusion in the multivariable model were restricted to those with established prior evidence of association with no-reflow or microvascular dysfunction in the ACS literature, namely, age, sex, diabetes mellitus, hypertension, and pre-procedural TIMI Flow Grade. This conservative variable selection was additionally guided by the events-per-variable (EPV) principle: with 46 no-reflow events, a maximum of four to five predictors could be reliably supported without risking model overfitting, consistent with the recommended EPV threshold of at least ten events per variable. Detailed procedural data, including intracoronary vasodilator use, glycoprotein IIb/IIIa inhibitor administration, thrombus aspiration, stent characteristics, and angiographic thrombus burden, were not systematically collected as part of the prospective data acquisition protocol, and their omission is acknowledged as a limitation of this study. Multicollinearity was assessed using the variance inflation factor (VIF) within each model separately; values <5 were considered acceptable. Because the three nutritional indices share overlapping components, they were evaluated in separate parallel models (M4A, M4B, M4C) rather than simultaneously, to avoid induced multicollinearity by design.

To quantify the incremental predictive value of each nutritional index, hierarchical binary logistic regression was performed across six sequentially constructed models: M1 (age and sex); M2 (M1 + diabetes mellitus and hypertension); M3 (M2 + pre-procedural TIMI Flow Grade); M4A (M3 + HALP score); M4B (M3 + PNI score); and M4C (M3 + CONUT score). Models M4A, M4B, and M4C represent parallel comparisons, each evaluated against M3 as the reference clinical model. Model discrimination was assessed by the area under the ROC curve (AUC), overall goodness-of-fit by the Akaike Information Criterion (AIC), and explanatory power by the Nagelkerke pseudo-R^2^. The incremental contribution of each nutritional index was formally tested using the likelihood-ratio (LR) χ^2^ test comparing each M4 model against M3.

Pairwise comparisons of AUC values across Models 4A, 4B, and 4C were performed using the DeLong method, which accounts for the correlation between ROC curves estimated on the same dataset.

Optimal cut-off values for each nutritional index were derived from individual ROC curves using the Youden Index (sensitivity + specificity − 1). Sensitivity, specificity, positive predictive value (PPV), negative predictive value (NPV), and likelihood ratios were calculated at each optimal threshold (Supplementary Table S1).

The incremental reclassification performance of each nutritional index beyond M3 was quantified using continuous Net Reclassification Improvement (NRI) and Integrated Discrimination Improvement (IDI). Bootstrap 95% confidence intervals for NRI and IDI were derived from 2000 resampling iterations using the percentile method (Supplementary Table S2).

Internal validation of Model 4C (the best-performing model) was performed using bootstrap resampling with 500 iterations. The bootstrap-corrected AUC, calibration intercept, calibration slope, and Brier score were calculated to assess discrimination, calibration, and overall predictive accuracy, respectively (Supplementary Table S6).

Decision curve analysis (DCA) was performed to evaluate the net clinical benefit of each nutritional model (M4A, M4B, M4C) relative to M3, the treat-all, and the treat-none strategies across a range of threshold probabilities from 1% to 50%.

### 2.7. Use of Artificial Intelligence

All scientific content, data analysis, interpretation, and final manuscript decisions were independently performed and approved by the authors. No AI tool was used in study design, data collection, or statistical analysis.

## 3. Results

### 3.1. Baseline Characteristics

A total of 279 patients were prospectively enrolled; 233 (83.5%) had an uneventful procedural course and 46 (16.5%) developed no-reflow during percutaneous coronary intervention. The mean age was 58.9 ± 12.1 years, and 183 patients (65.6%) were male. Rates of diabetes mellitus (30.8%), hypertension (48.4%), and current or ex-smoking (58.4%) were comparable between groups (all *p* > 0.05). STEMI accounted for 35.1% of presentations and NSTEMI for 64.9%; no-reflow occurred in 17.3% of STEMI and 16.0% of NSTEMI patients, with no significant difference between presentation types (*p* = 0.908). The culprit vessel was the left anterior descending artery in 43.0%, the circumflex in 30.1%, and the right coronary artery in 26.5% of cases; the distribution of culprit artery did not differ significantly between no-reflow and non–no-reflow groups (all *p* > 0.05). Further demographic and clinical details are provided in Table 1 and Supplementary Table S5.

### 3.2. Nutritional Status and No-Reflow

Lower HALP and PNI scores and higher CONUT scores were observed among patients who developed no-reflow (all *p* < 0.001). The HALP score, PNI score, and CONUT score each showed a statistically significant difference between patients with and without no-reflow (all *p* < 0.001; Table 1). TIMI Flow Grade was the only clinical procedural variable that differed significantly between groups (*p* = 0.004), whereas albumin, platelet count, triglyceride, LDL-cholesterol, and total cholesterol also differed at the individual laboratory level (Table 1).

Spearman rank correlation analysis confirmed associations between no-reflow and all three nutritional indices: CONUT score (ρ = +0.322), PNI score (ρ = −0.275), and HALP score (ρ = −0.206) each reached *p* < 0.001, whereas TIMI Flow Grade yielded ρ = −0.175 (*p* = 0.003). None of the demographic or comorbidity variables correlated significantly with no-reflow occurrence (Table 2).

### 3.3. Univariate and Multivariable Logistic Regression

On univariate logistic regression, TIMI Flow Grade (OR 0.545, 95% CI 0.357–0.832, *p* = 0.005), HALP score (OR 0.960, 95% CI 0.938–0.982, *p* < 0.001), PNI score (OR 0.862, 95% CI 0.807–0.921, *p* < 0.001), and CONUT score (OR 2.015, 95% CI 1.548–2.622, *p* < 0.001) were each associated with no-reflow; no demographic or comorbidity variable reached significance. In the forced-entry multivariable model including all univariate predictors, only CONUT score retained independent significance (OR 1.728, 95% CI 1.226–2.435, *p* = 0.002; Nagelkerke R^2^ = 0.153). All variance inflation factors were below 1.3, confirming the absence of multicollinearity (Table 3; Supplementary Table S3).

**Table 3:** Logistic Regression Analysis—Univariate and Multivariable Models.

Variable	Univariate Analysis			Multivariable Analysis		
	OR	95% CI	*p*	OR	95% CI	*p*
Demographic and Clinical Covariates						
Age, per year	1.001	0.975–1.027	0.951	—	—	—
Sex, male	1.827	0.882–3.785	0.105	—	—	—
Smoking	1.014	0.533–1.926	0.967	—	—	—
Diabetes mellitus	1.104	0.561–2.172	0.774	—	—	—
Hypertension	1.080	0.574–2.034	0.811	—	—	—
Pre-procedural TIMI Flow Grade	0.545	0.357–0.832	0.005	0.646	0.399–1.047	0.076
Presentation (STEMI)	1.100	0.570–2.121	0.776	—	—	—
Nutritional Status Indices						
HALP Score	0.960	0.938–0.982	<0.001	0.987	0.959–1.015	0.356
PNI Score	0.862	0.807–0.921	<0.001	0.971	0.882–1.069	0.550
CONUT Score	2.015	1.548–2.622	<0.001	1.728	1.226–2.435	0.002

Variables with *p* < 0.05 on univariate analysis were entered into the multivariable model using a forced-entry approach. Reference category for binary variables: absent/female. OR, odds ratio; 95% CI, confidence interval. Variables with *p* < 0.05 on univariate analysis were entered into the multivariable model. **Model statistics:** Nagelkerke R^2^ = 0.153, AIC = 221.6. OR, odds ratio; CI, confidence interval; —, not entered into multivariable model. HALP, PNI, and CONUT were entered simultaneously into the forced-entry multivariable model to assess independent association under mutual adjustment. This is distinct from the parallel hierarchical models in Table 4 (M4A, M4B, M4C), where each index was evaluated separately to quantify incremental discriminative value without inducing multicollinearity.

**Table 4:** Hierarchical Binary Logistic Regression—Sequential Model Building.

Model	Covariates	Model Fit Statistics			LR Test vs. Previous Model
		AIC	R^2^	AUC	
M1	Age + Sex	252.9	0.012	0.568	Reference
M2	M1 + DM + HT	256.6	0.013	0.581	χ^2^ = 0.31, *p* = 0.859
M3	M2 + Pre-procedural TIMI Flow Grade	249.6	0.049	0.649	χ^2^ = 8.96, *p* = 0.003
M4A	M3 + HALP Score	240.0	0.095	0.693	χ^2^ = 11.62, *p* = 0.001
M4B	M3 + PNI Score	234.3	0.118	0.733	χ^2^ = 17.31, *p* < 0.001
M4C	M3 + CONUT Score	225.6	0.153	0.770	χ^2^ = 25.98, *p* < 0.001

OR values represent odds ratios (binary logistic regression); *p* values are shown in parentheses. Models M4A, M4B, and M4C each add one nutritional index to M3 and are compared against M3 via likelihood-ratio (LR) χ^2^ test. Abbreviations: DM, diabetes mellitus; HT, hypertension; TIMI, Thrombolysis in Myocardial Infarction; HALP, Hemoglobin–Albumin–Lymphocyte–Platelet score; PNI, Prognostic Nutritional Index; CONUT, Controlling Nutritional Status score; R^2^, Nagelkerke pseudo-R^2^; AUC, area under the ROC curve; LR, likelihood ratio.

### 3.4. Hierarchical Model Building and Discrimination

**Figure 2:**
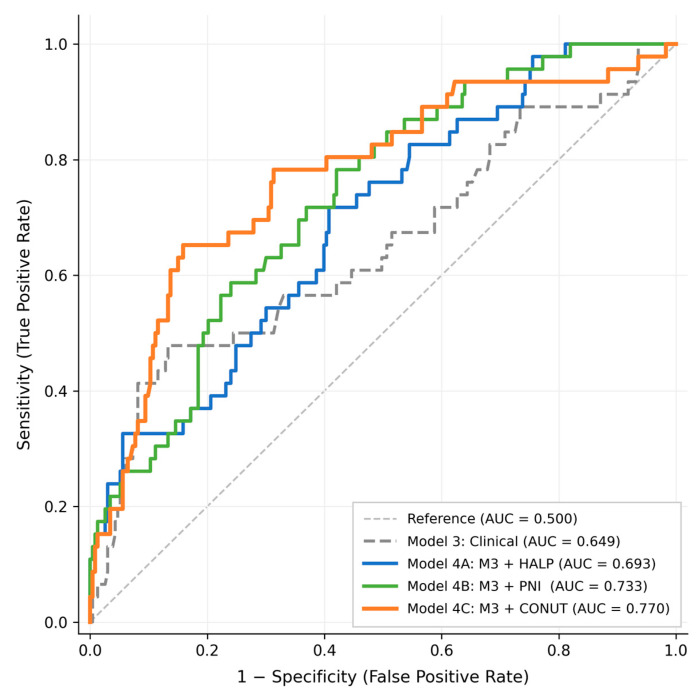
Receiver Operating Characteristic Curves of Hierarchical Prediction Models for No-Reflow. Receiver operating characteristic (ROC) curves comparing the baseline clinical model (Model 3: age, sex, diabetes mellitus, hypertension, and TIMI Flow Grade with models additionally incorporating HALP (Model 4A), PNI (Model 4B), and CONUT (Model 4C) scores. The addition of nutritional indices improved model discrimination for no-reflow prediction. Areas under the curve (AUCs) were 0.649 for Model 3, 0.693 for Model 4A, 0.733 for Model 4B, and 0.770 for Model 4C. The diagonal reference line represents an AUC of 0.50. Abbreviations: HALP, hemoglobin–albumin–lymphocyte–platelet score; PNI, Prognostic Nutritional Index; CONUT, controlling nutritional status score.

Sequential hierarchical modelling was performed to quantify the incremental value of each nutritional index beyond clinical covariates. The base demographic model (M1: age and sex; AUC 0.568) was not significantly improved by the addition of diabetes mellitus and hypertension (M2: AUC 0.581, LR χ^2^ = 0.31, *p* = 0.859). Adding TIMI Flow Grade (M3) produced a significant improvement in discrimination (AUC 0.649, LR χ^2^ = 8.96, *p* = 0.003). Each nutritional index was then evaluated as a separate additive term in M3. HALP score (M4A) raised the AUC to 0.693 (LR χ^2^ = 11.62, *p* = 0.001), PNI score (M4B) to 0.733 (LR χ^2^ = 17.31, *p* < 0.001), and CONUT score (M4C) to 0.770 (LR χ^2^ = 25.98, *p* < 0.001; Table 4; Figure 2).

Pairwise DeLong comparisons of M4A, M4B, and M4C revealed no statistically significant differences in AUC between the three nutritional models (all *p* > 0.05), although the comparison of M4A versus M4C showed a trend favouring CONUT (ΔAUC = 0.077, *p* = 0.055; Supplementary Table S4). CONUT achieved the largest absolute likelihood-ratio gain over M3 (χ^2^ = 25.98) relative to HALP (χ^2^ = 11.62) and PNI (χ^2^ = 17.31). Accordingly, these numerical differences should not be interpreted as definitive evidence of superiority.

### 3.5. Optimal Cut-Offs and Reclassification

Youden index optimal cut-offs were identified for each index from individual ROC curves (Supplementary Figure S1). A CONUT score ≥2 yielded the highest Youden index (0.439), sensitivity (78.3%), specificity (65.7%), and negative predictive value (93.9%), with a negative likelihood ratio of 0.331. Categorical analysis further demonstrated a stepwise increase in no-reflow rates across CONUT nutritional classes: 6.1% among patients with normal nutritional status (CONUT 0–1, *n* = 163), 28.8% among those with mild malnutrition (CONUT 2–4, *n* = 111), and 80.0% among those with moderate malnutrition (CONUT 5–8, *n* = 5). Patients with CONUT ≥ 2 faced nearly seven-fold higher odds of no-reflow compared with those with normal nutritional status (OR 6.885, 95% CI 3.249–14.590, *p* < 0.001; Supplementary Table S7). Corresponding thresholds were ≤38.25 for HALP (Youden index 0.275) and ≤48.10 for PNI (Youden index 0.335; Supplementary Table S1).

Adding CONUT score to M3 produced a continuous net reclassification improvement of 0.757 (95% CI 0.464–1.118, *p* < 0.001) and an integrated discrimination improvement of 0.104 (95% CI 0.037–0.206, *p* < 0.001), reflecting a 8.7 percentage-point increase in mean predicted risk among patients who developed no-reflow. The corresponding NRI and IDI for HALP and PNI were significant but numerically smaller (Supplementary Table S2).

### 3.6. Clinical Utility and Internal Validation

**Figure 3:**
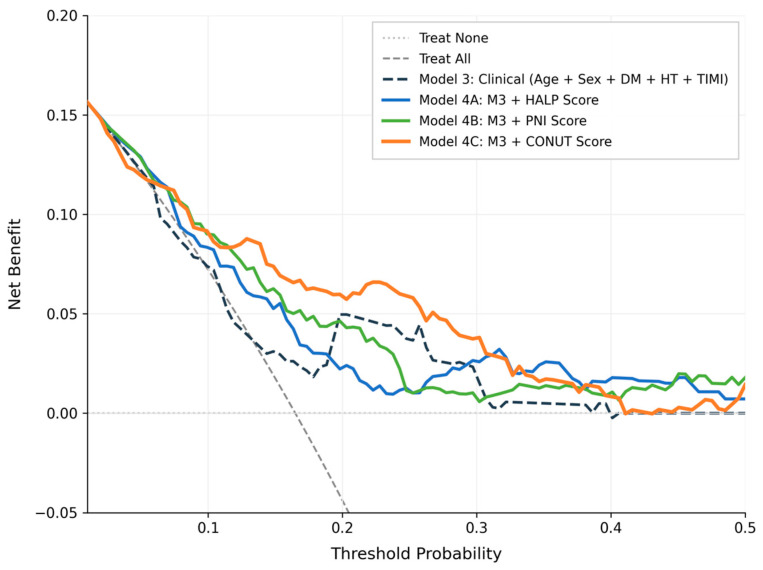
Decision Curve Analysis of Hierarchical Prediction Models for No-Reflow Development. Decision curve analysis comparing the net clinical benefit of the baseline clinical model (Model 3: age, sex, diabetes mellitus, hypertension, and TIMI Flow Grade) with models additionally incorporating HALP (Model 4A), PNI (Model 4B), and CONUT (Model 4C) scores. The horizontal dotted line represents the strategy of treating no patients, whereas the dashed grey line represents treating all patients. Across a range of threshold probabilities, the nutritional models generally demonstrated a higher net benefit than the clinical model alone, with the greatest net benefit observed for Model 4C (CONUT). Abbreviations: HALP, hemoglobin–albumin–lymphocyte–platelet score; PNI, Prognostic Nutritional Index; CONUT, controlling nutritional status score.

Decision curve analysis demonstrated that Model 4C provided greater net clinical benefit than the clinical model alone across a threshold probability range of approximately 15–30%, and consistently outperformed both the treat-all and treat-none strategies within this range (Figure 3). Internal bootstrap validation (500 iterations) of Model 4C yielded a corrected AUC of 0.754 (95% CI 0.725–0.768), a calibration intercept of 0.046, a calibration slope of 1.034, and a Brier score of 0.116, indicating good predictive accuracy and minimal optimism (Supplementary Table S6).

## 4. Discussion

### 4.1. Principal Findings

In this prospective study of 279 patients with ACS undergoing PCI, no-reflow occurred in 46 patients (16.5%), a rate consistent with contemporary series in mixed STEMI–NSTEMI populations [11]. All three nutritional indices, HALP, PNI, and CONUT, were significantly associated with no-reflow on univariate analysis and in Spearman correlation, whereas none of the conventional demographic or comorbidity variables reached significance. In multivariable analysis, only the CONUT score retained statistical significance after adjustment for the available covariates. Hierarchical modelling demonstrated that each nutritional index improved discrimination beyond a clinical reference model incorporating age, sex, comorbidities, and pre-procedural TIMI Flow Grade, with the largest likelihood-ratio gain observed for CONUT (LR χ^2^ = 25.98 vs. 17.31 for PNI and 11.62 for HALP). DeLong pairwise comparisons, however, did not reveal statistically significant differences among the three nutritional models, and this pattern should be interpreted with appropriate caution given the modest event count.

### 4.2. Comparison with Previous Studies

The 16.5% no-reflow rate observed in our cohort falls within the range reported across contemporary ACS studies, where incidence varies with patient selection, procedural context, and diagnostic criteria [13,14]. Reported no-reflow rates in contemporary ACS cohorts generally range between 10% and 20%, consistent with the incidence observed in our study [14].

The association between nutritional indices and mortality or major adverse cardiovascular events in ACS populations is well established. A meta-analysis of 37,303 patients reported that malnutrition was independently associated with all-cause mortality, regardless of ACS type or region [15]. The CONUT score specifically has been linked to MACE and mortality in patients with ACS and myocardial infarction across multiple studies and a recent meta-analysis [9,16]. Similarly, PNI has demonstrated consistent prognostic value in ACS cohorts undergoing PCI [8]. For the HALP score, prior work has confirmed its association with no-reflow and short-term mortality in STEMI patients undergoing primary PCI [10,17].

This study extends these observations in several ways. First, unlike most prior reports that evaluated a single nutritional index, we directly compared all three indices within the same prospective cohort. Second, our population included both STEMI and NSTEMI patients, reflecting a broader clinical spectrum than STEMI-only series. Third, we applied a comprehensive analytical framework encompassing hierarchical modelling, reclassification statistics, DeLong testing, decision curve analysis, and bootstrap internal validation, enabling a more complete characterisation of each index’s incremental value. A comparable multitool comparison of nutritional scores, albeit for MACE prediction rather than no-reflow, was recently reported in elderly ACS patients after PCI, where PNI showed numerically superior discrimination among four indices evaluated by similar methods [18]. Our findings align with that report in demonstrating that the indices are broadly complementary and that definitive ranking requires caution.

Notably, despite the numerically superior performance of CONUT across several metrics, formal pairwise DeLong comparisons did not demonstrate statistically significant superiority over HALP or PNI, suggesting that the observed differences should be interpreted cautiously.

### 4.3. Potential Mechanisms

The biological pathways linking impaired nutritional status to microvascular dysfunction are likely to be multifactorial. Albumin, which contributes to all three indices, carries well-characterised anti-inflammatory, antioxidant, anticoagulant, and antiplatelet properties [19]. It also plays a central role in maintaining endothelial glycocalyx integrity, a key determinant of microvascular homeostasis through transport of sphingosine-1-phosphate and free radical scavenging activity [20]. Hypoalbuminaemia may therefore amplify endothelial vulnerability and the inflammatory cascade triggered by ischaemia–reperfusion injury.

Lymphocyte count, incorporated in both HALP and PNI, reflects immune competence and systemic inflammatory burden. Lymphopenia following myocardial infarction has been associated with larger infarct size, higher troponin levels, and microvascular obstruction on cardiac magnetic resonance imaging [21,22]. The inverse relationship between lymphocyte count and no-reflow is therefore mechanistically plausible and consistent with the broader literature on immune-mediated microvascular injury.

The numerically stronger performance of CONUT in this study may relate to its incorporation of total cholesterol alongside albumin and lymphocyte count. In the acute phase of myocardial infarction, total cholesterol may decline as part of the inflammatory response and hepatic acute-phase protein reprioritisation. Under these conditions, low total cholesterol may reflect not only nutritional depletion but also the magnitude of systemic inflammation and metabolic stress, dimensions not fully captured by HALP or PNI [9]. This interpretation remains speculative, and this study was not designed to investigate causal pathways.

### 4.4. Clinical Implications

All three nutritional indices are calculable from routine admission laboratory parameters without additional cost, time, or testing. Their availability at the point of care makes them attractive candidates for simple, adjunctive risk stratification in patients undergoing coronary intervention. The decision curve analysis demonstrated that all three nutritional models provided greater net clinical benefit than the clinical model alone across threshold probabilities of approximately 15–30%, suggesting potential relevance for guiding preventive strategies in patients at intermediate to high procedural risk.

These findings should be interpreted conservatively. The results indicate that nutritional status is associated with no-reflow susceptibility and that nutritional indices may provide information complementary to conventional clinical variables. They do not establish that nutritional scores should replace or supersede existing risk tools, nor do they support their use in isolation. External validation in larger, independent cohorts is required before any recommendation for clinical implementation can be considered. Whether nutritional optimisation strategies are capable of modifying no-reflow risk is a question that lies beyond the scope of this study and warrants prospective investigation.

### 4.5. Limitations

Several important procedural and clinical determinants of no-reflow were not systematically collected as part of the study protocol. These included angiographic thrombus burden, distal embolisation, intracoronary pharmacotherapy, glycoprotein IIb/IIIa inhibitor use, stent length and diameter, lesion complexity, post-dilatation strategy, and detailed procedural characteristics. Furthermore, markers of infarct severity and myocardial injury, including peak cardiac biomarker levels, infarct size, microvascular obstruction, and left ventricular systolic function, were unavailable for adjustment. Because these factors are strongly associated with no-reflow occurrence and may also be linked to nutritional status, residual confounding cannot be excluded despite multivariable adjustment.

First, this was a single-centre investigation, and the generalisability of the findings to other populations, healthcare settings, or ethnic groups cannot be assumed. External validation in independent, multicentre cohorts with larger event numbers is necessary before broader conclusions can be drawn.

Second, no-reflow was defined on angiographic criteria post-procedural TIMI flow grade and myocardial blush grade. More sensitive assessments of microvascular dysfunction, including cardiac magnetic resonance imaging with late gadolinium enhancement or invasive index of microcirculatory resistance measurement, were not available. Some degree of outcome misclassification is therefore possible, and subclinical microvascular injury may have been underdetected.

Third, nutritional status was characterised from a single blood sample obtained at the time of admission. Distinguishing chronic malnutrition from acute metabolic alterations related to the ischaemic event itself, including the acute-phase response and its effects on albumin and cholesterol, was not possible with the available data. Detailed dietary evaluation, body-composition analysis, and validated nutritional screening tools were not applied.

Fourth, the number of clinical covariates incorporated into the multivariable models was intentionally limited by two considerations: the available events-per-variable ratio given 46 no-reflow events, and the prospective data acquisition protocol, which did not include systematic collection of several important procedural and clinical determinants of no-reflow. These included angiographic thrombus burden, distal embolisation, intracoronary pharmacotherapy, glycoprotein IIb/IIIa inhibitor use, thrombus aspiration, stent length and diameter, lesion complexity, inflation pressure, post-dilatation strategy, and other procedural characteristics. Furthermore, markers of infarct severity and myocardial injury, including peak cardiac biomarker concentrations, infarct size, microvascular obstruction, left ventricular ejection fraction, and other measures of ventricular function, were unavailable for adjustment. Because these factors are strongly associated with no-reflow occurrence and may also be linked to nutritional status, residual confounding cannot be excluded, despite multivariable adjustment. Their incorporation into future prospective studies with dedicated procedural and imaging data collection would further strengthen the robustness of nutritional-index-based risk models.

Fifth, although the sample size of 279 patients yielded 46 no-reflow events, the absolute event count remained modest and may have limited the statistical power to detect smaller differences between nutritional indices. The absence of significant differences in pairwise DeLong comparisons should therefore be interpreted in this context.

In addition, patients with previous coronary revascularisation and those with significant left main coronary artery disease were excluded to maintain a more homogeneous study population. Consequently, the applicability of these findings to more complex coronary disease subsets should be interpreted with caution.

Furthermore, the upper age limit of 70 years applied in the inclusion criteria excluded a substantial proportion of real-world ACS patients. Older patients represent approximately one-third of ACS presentations and carry a disproportionately higher burden of both malnutrition and microvascular dysfunction, rendering them particularly relevant to the research question addressed in this study. Their exclusion may therefore limit the generalisability of these findings to elderly populations and may have influenced the observed magnitude of association of nutritional indices on no-reflow susceptibility. Readers should exercise caution when extrapolating these results to patients older than 70 years, and future studies specifically enrolling elderly ACS cohorts are warranted.

Finally, the study focused on the angiographic endpoint of no-reflow and did not include longitudinal clinical follow-up. The relationship between nutritional-index-associated no-reflow and hard outcomes including mortality, heart failure, and recurrent ischaemic events remains to be characterised in studies with extended follow-up.

Importantly, this study was not designed to determine whether nutritional impairment directly contributes to the pathogenesis of no-reflow. The observed associations may reflect the complex interplay between nutritional status, systemic inflammation, metabolic disturbances, infarct severity, and procedural characteristics. Therefore, our findings should be interpreted as demonstrating an association rather than a causal relationship. These results suggest that routinely available nutritional indices may help identify patients at increased risk of no-reflow; however, dedicated mechanistic and interventional studies are required before any causal inference can be made.

Consequently, the observed associations between nutritional indices and no-reflow should be interpreted with caution. Although nutritional indices improved model performance and remained associated with no-reflow across several analytical approaches, the possibility that they partially capture unmeasured disease severity, inflammatory burden, or procedural complexity cannot be excluded. Therefore, these findings should be considered hypothesis-generating and require confirmation in larger multicentre studies incorporating comprehensive clinical, angiographic, imaging, and procedural data.

## 5. Conclusions

This prospective study addressed two pre-specified aims. All three nutritional indices were significantly associated with no-reflow in univariate analyses and improved model discrimination when evaluated separately within hierarchical models. After adjustment in the multivariable model, only CONUT remained statistically significant. With respect to the second, CONUT demonstrated the most consistent performance across multivariable, discrimination, and reclassification analyses: a CONUT score ≥ 2, corresponding to the threshold between normal nutritional status and mild-to-moderate malnutrition, yielded the highest Youden index (0.439), the largest likelihood-ratio gain over the clinical model (χ^2^ = 25.98), and the greatest net reclassification improvement (NRI 0.757) and integrated discrimination improvement (IDI 0.104) among the three indices. Statistically significant superiority over HALP and PNI was not established in pairwise DeLong comparisons, a finding that should be interpreted in the context of the modest event count. Corresponding optimal thresholds were a HALP score ≤ 38.25 and a PNI score ≤ 48.10, both indicative of nutritional impairment at the point of admission.

Taken together, these findings indicate that admission nutritional status—readily assessable from routine laboratory parameters without additional cost or delay—is closely related to susceptibility to no-reflow and may provide clinically meaningful complementary information for procedural risk stratification in ACS. Larger, multicentre prospective studies with extended clinical follow-up are needed to externally validate these observations, refine optimal thresholds across diverse populations, and determine whether targeted nutritional intervention prior to or following PCI is capable of modifying no-reflow risk. These findings should be considered hypothesis-generating and require external validation in larger multicentre ACS cohorts.

## Figures and Tables

**Table 1:** Baseline Clinical Characteristics.

Variable	Total (*n* = 279)	No-Reflow (−) (*n* = 233, 83.5%)	No-Reflow (+) (*n* = 46, 16.5%)	*p* Value
Demographics				
Age (years)	58.9 ± 12.1	58.8 ± 12.4	59.0 ± 10.8	0.915
Male sex, *n* (%)	183 (65.6%)	148 (63.5%)	35 (76.1%)	0.142
Smoking, *n* (%)	163 (58.4%)	136 (58.4%)	27 (58.7%)	1.000
Comorbidities				
Diabetes mellitus, *n* (%)	86 (30.8%)	71 (30.5%)	15 (32.6%)	0.911
Hypertension, *n* (%)	135 (48.4%)	112 (48.1%)	23 (50.0%)	0.938
Clinical Presentation				
STEMI, *n* (%)	98 (35.1%)	81 (34.8%)	17 (37.0%)	0.908
NSTEMI, *n* (%)	181 (64.9%)	152 (65.2%)	29 (63.0%)	0.908
Pre-procedural TIMI Flow Grade	1.0 (1.0–2.0)	1.0 (1.0–2.0)	1.0 (0.0–1.8)	0.004
Culprit Coronary Artery				
LAD, *n* (%)	120 (43.0%)	103 (44.2%)	17 (37.0%)	0.457
CX, *n* (%)	84 (30.1%)	66 (28.3%)	18 (39.1%)	0.199
RCA, *n* (%)	74 (26.5%)	62 (26.6%)	12 (26.1%)	1.000
Nutritional Indices				
HALP Score	40.7 [30.9–53.4]	41.4 [31.9–56.1]	34.4 [25.5–44.1]	*<0.001*
PNI Score	48.9 [45.0–53.0]	49.8 [45.7–53.8]	45.2 [41.9–48.7]	*<0.001*
CONUT Score	1.0 [0.0–2.0]	1.0 [0.0–2.0]	2.0 [2.0–3.0]	*<0.001*
Laboratory Parameters				
Hemoglobin (g/dL)	13.6 [12.1–14.8]	13.7 [12.3–14.8]	12.8 [11.5–14.5]	0.036
Lymphocyte (×10^3^/µL)	2.1 [1.7–2.7]	2.0 [1.7–2.6]	2.4 [1.7–2.8]	0.338
Albumin (g/dL)	3.9 [3.4–4.2]	4.0 [3.6–4.2]	3.3 [3.2–3.6]	*<0.001*
Platelet (×10^3^/µL)	271 [223.5–326.0]	268 [215–320]	298 [255.8–358.2]	0.008
Total Cholesterol (mg/dL)	183.2 [159.8–218.1]	181.6 [160.6–213.2]	216.6 [154.1–242.1]	0.034
HDL-C (mg/dL)	45.0 [39–52]	45.0 [40–53]	43.0 [39.2–49.0]	0.161
LDL-C (mg/dL)	108 [90.5–134.5]	107 [91–132]	131 [90.8–156.2]	0.021
Triglyceride (mg/dL)	125 [99.5–193.0]	123 [98–178]	164.5 [112.2–226.2]	0.010

Continuous variables are presented as mean ± standard deviation (SD) for normally distributed variables and as median (interquartile range [IQR]) for non-normally distributed variables. Group comparisons were performed using the independent samples *t*-test or Mann–Whitney U test, as appropriate. Categorical variables are presented as n (%) and compared using Pearson’s χ^2^ test or Fisher’s exact test when appropriate. A two-sided *p* value < 0.05 was considered statistically significant. **Abbreviations:** DM, diabetes mellitus; HT, hypertension; STEMI, ST-elevation myocardial infarction; NSTEMI, non-ST-elevation myocardial infarction; LAD, left anterior descending artery; CX, circumflex artery; RCA, right coronary artery; TIMI, Thrombolysis in Myocardial Infarction; HALP, Hemoglobin–Albumin–Lymphocyte–Platelet score; PNI, Prognostic Nutritional Index; CONUT, Controlling Nutritional Status score; HDL-C, high-density lipoprotein cholesterol; LDL-C, low-density lipoprotein cholesterol.

**Table 2:** Spearman Rank Correlation—No-Reflow with Clinical and Nutritional Parameters.

Variable	Spearman ρ	*p* Value	Direction of Effect
Demographic and Clinical Covariates			
Age	+0.006	0.914	
Sex (male)	+0.098	0.102	
Smoking	+0.002	0.967	
Diabetes mellitus	+0.017	0.775	
Hypertension	+0.014	0.811	
Clinical Presentation			
Pre-procedural TIMI Flow Grade	−0.175	0.003	Higher TFG → lower no-reflow risk (higher flow grade reflects better baseline coronary perfusion)
STEMI	+0.017	0.777	
Nutritional Status Indices			
HALP Score	−0.206	<0.001	Nutritional index; inversely associated
PNI Score	−0.275	<0.001	Nutritional index; inversely associated
CONUT Score	+0.322	<0.001	Nutritional index; positively associated

Spearman ρ coefficients between each predictor and no-reflow occurrence (binary). No imputation was required (complete case, *n* = 279). Significant correlations (*p* < 0.05) are highlighted in bold. **Abbreviations:** TFG, TIMI Flow Grade; HALP, Hemoglobin–Albumin–Lymphocyte–Platelet score; PNI, Prognostic Nutritional Index; CONUT, Controlling Nutritional Status score.

## Data Availability

The data supporting the findings of this study are available from the corresponding author upon reasonable request.

## Supplementary Materials

The following supporting information can be downloaded at: https://www.mdpi.com/article/10.3390/jcm15135191/s1, Supplementary Figure S1: Receiver operating characteristic (ROC) curves and Youden optimal cut-off analyses for HALP, PNI, and CONUT scores; Supplementary Table S1: Optimal cut-off analysis and diagnostic performance metrics; Supplementary Table S2: Net Reclassification Improvement (NRI) and Integrated Discrimination Improvement (IDI) analyses; Supplementary Table S3: Variance Inflation Factor (VIF) assessment of multicollinearity; Supplementary Table S4: Pairwise DeLong comparisons of model discrimination; Supplementary Table S5: No-reflow rates according to ACS presentation type and culprit vessel; Supplementary Table S6: Internal Validation and Calibration of Model 4C; Supplementary Table S7: No-reflow rates and odds ratios stratified by CONUT score nutritional category.

## Abbreviations

ACS	Acute coronary syndrome
AIC	Akaike Information Criterion
AUC	Area under the receiver operating characteristic curve
CI	Confidence interval
CONUT	Controlling Nutritional Status score
CX	Circumflex artery
DCA	Decision curve analysis
DM	Diabetes mellitus
ESC	European Society of Cardiology
HALP	Hemoglobin–Albumin–Lymphocyte–Platelet score
HDL-C	High-density lipoprotein cholesterol
HT	Hypertension
IDI	Integrated Discrimination Improvement
IQR	Interquartile range
LAD	Left anterior descending artery
LDL-C	Low-density lipoprotein cholesterol
LR	Likelihood ratio
MACE	Major adverse cardiovascular events
MBG	Myocardial blush grade
NPV	Negative predictive value
NRI	Net Reclassification Improvement
NSTEMI	Non-ST-elevation myocardial infarction
OR	Odds ratio
PCI	Percutaneous coronary intervention
PNI	Prognostic Nutritional Index
PPV	Positive predictive value
RCA	Right coronary artery
ROC	Receiver operating characteristic
SD	Standard deviation
STEMI	ST-elevation myocardial infarction
TFG	TIMI Flow Grade
TIMI	Thrombolysis in Myocardial Infarction
VIF	Variance inflation factor
