# Supplementary material for: Comparative Performance of HALP, PNI, and CONUT Scores in No-Reflow Among Patients with Acute Coronary Syndrome: A Prospective Study
Source: J Clin Med. 2026 Jul 2;15(13):5191. doi: 10.3390/jcm15135191 (PMC13362716; doi:10.3390/jcm15135191)
Supplement: Supplementary file 1 [file jcm-15-05191-s001.zip › jcm-4371418-supplementary.pdf]

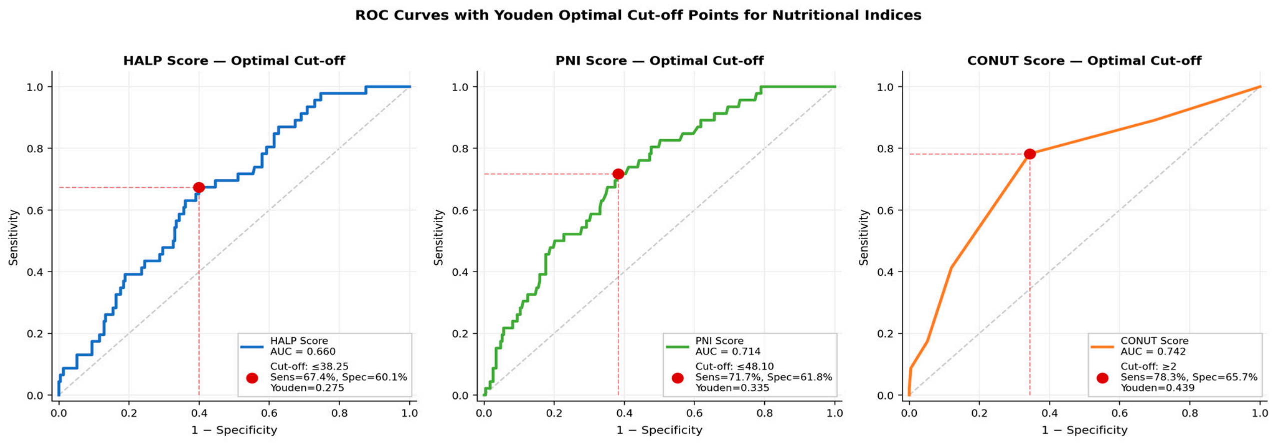

Supplementary Figure S1. ROC curves with Youden optimal cut-off points for HALP Score (≤38.25), PNI Score (≤48.10) and CONUT Score (≥2). Red circles mark the optimal operating point; dashed lines project sensitivity and specificity to the axes.

Supplementary Table S1. Optimal Cut-off Analysis — HALP, PNI and CONUT Scores (Youden Index)

Parameter	HALP Score	PNI Score	CONUT Score
AUC (index alone)	0.660	0.714	**0.742**
Optimal cut-off	≤ 38.25	≤ 48.10	**≥ 2**
Direction of risk	Lower = worse	Lower = worse	**Higher = worse**
Sensitivity	67.4%	71.7%	**78.3%**
Specificity	60.1%	61.8%	**65.7%**
Youden Index	0.275	0.335	**0.439**
PPV	25.0%	27.0%	**31.0%**
NPV	90.3%	91.7%	**93.9%**
Positive LR (LR+)	1.688	1.878	**2.279**
Negative LR (LR−)	0.543	0.457	**0.331**

Optimal operating points were identified from individual ROC curves using the Youden Index (sensitivity + specificity − 1). For HALP and PNI, lower values confer higher no-reflow risk (inverse direction). PPV and NPV are prevalence-dependent (no-reflow rate = 16.5%).

AUC, area under ROC curve; PPV, positive predictive value; NPV, negative predictive value; LR+/LR−, positive/negative likelihood ratio. Bold column = best performing index across all metrics.

Supplementary Table S2. Net Reclassification Improvement (NRI) and Integrated Discrimination Improvement (IDI)

Metric	M4A (M3+HALP Score)			M4B (M3+PNI Score)			M4C (M3+CONUT Score)		
	Est.	95% CI	p	Est.	95% CI	p	Est.	95% CI	p
**Continuous NRI**	**0.542**	0.195–0.895	**0.001**	**0.584**	0.265–0.939	**<0.001**	**0.757**	0.464–1.118	**<0.001**
NRI (events)	0.391	—	—	0.348	—	—	0.435	—	—
NRI (non-events)	0.150	—	—	0.236	—	—	0.322	—	—
**IDI**	**0.038**	0.007–0.097	**0.005**	**0.058**	0.017–0.130	**0.001**	**0.104**	0.037–0.206	**<0.001**

Continuous NRI and IDI quantify incremental predictive gain of each nutritional index over Model 3 (Age + Sex + DM + HT + TIMI Frame Rate). Bootstrap 95% CIs based on 2 000 resamples (percentile method).

NRI, Net Reclassification Improvement; IDI, Integrated Discrimination Improvement; Est., point estimate. Bootstrap 95% CI shown for primary metrics only.

Supplementary Table S3. Variance Inflation Factor (VIF) — Multicollinearity Assessment

Variable	Model 4A (M3 + HALP)		Model 4B (M3 + PNI)		Model 4C (M3 + CONUT)	
	VIF	Interp.	VIF	Interp.	VIF	Interp.
Age	1.162	None	1.166	None	1.125	None
Male sex	1.061	None	1.055	None	1.055	None
Diabetes mellitus	1.270	None	1.268	None	1.268	None
Hypertension	1.206	None	1.197	None	1.192	None
TIMI Frame Rate	1.208	None	1.129	None	1.082	None
HALP Score	1.242	None	—	—	—	—
PNI Score	—	—	1.140	None	—	—
CONUT Score	—	—	—	—	1.049	None

VIF <2: negligible collinearity; VIF 2–5: moderate; VIF >10: problematic. All values <1.3 confirm the absence of multicollinearity within each model. The note within the table explains why cross-index collinearity is not applicable.

VIF was assessed separately within each model (M4A, M4B, M4C). The three nutritional indices were **never entered simultaneously** *into the same regression model; accordingly, cross-index VIF is not applicable and not reported. Each model contains only one nutritional index alongside the same set of clinical covariates.*

Note: *The three nutritional indices (HALP, PNI, CONUT) were evaluated in separate models (M4A, M4B, M4C) and were never entered simultaneously into the same regression model. VIF values therefore reflect within-model collinearity only. Cross-index collinearity (e.g. HALP vs PNI) is not reported here because the indices are not co-modelled; this is by design to avoid redundancy and multicollinearity between overlapping composite scores.*

Supplementary Table S4. DeLong AUC Comparison — M4A vs M4B vs M4C

Comparison	AUC Model 1	AUC Model 2	ΔAUC	Z statistic	p value (DeLong)	Significance
**M4A vs M4B**	0.693 (HALP)	0.733 (PNI)	-0.040	-1.448	0.148	*ns*
**M4A vs M4C**	0.693 (HALP)	0.770 (CONUT)	-0.077	-1.921	0.055	*ns†*
**M4B vs M4C**	0.733 (PNI)	0.770 (CONUT)	-0.037	-1.379	0.168	*ns*

ΔAUC = AUC Model 1 − AUC Model 2 (negative value indicates Model 2 is superior). ns, not significant; †, p=0.055 (trend). DeLong Z statistic; two-tailed p value. Pairwise AUC comparisons were performed using the DeLong method (structural component approach), which accounts for the correlation between ROC curves estimated on the same dataset.

† *M4A vs M4C approaches but does not reach statistical significance (p=0.055). All three nutritional models show consistent direction of improvement over M3. CONUT provides numerically the highest AUC, and its LR χ^2 gain over M3 is the largest (χ^2=25.98 vs 11.62 for HALP and 17.31 for PNI).*

Supplementary Table S5. No-Reflow Rates by Presentation and Culprit Artery

Subgroup	n	No-Reflow (n)	No-Reflow Rate (%)	95% Wilson CI	Comment
**By Presentation Type**					
STEMI	98	17	17.3%	9.9–26.6%	
NSTEMI	181	29	16.0%	10.6–22.6%	
**By Culprit Coronary Artery**					
LAD	120	17	14.2%	8.4–22.1%	*Most common culprit*
CX	84	18	21.4%	13.1–32.2%	*Highest crude rate*
RCA	74	12	16.2%	8.7–26.6%	

Wilson 95% CI for proportions. Coronary artery groups are not mutually exclusive in cases of multivessel disease. No significant difference in no-reflow rate between STEMI and NSTEMI (χ^2=0.013, p=0.908), or between culprit artery groups.

Crude no-reflow rates with 95% Wilson confidence intervals across pre-specified subgroups. No nutritional index is included; this table describes the distribution of the outcome variable.

Supplementary Table S6. Internal Validation and Calibration of Model 4C

Metric	Value
Apparent AUC	0.770
Bootstrap-validated AUC	0.754
95% Bootstrap CI	0.725–0.768
Calibration intercept	0.046
Calibration slope	1.034
Brier score	0.116

Model performance was internally validated using bootstrap resampling (500 iterations). Discrimination was assessed by the bootstrap-corrected AUC, calibration by the calibration intercept and slope, and overall accuracy by the Brier score.

Supplementary Table S7. No-Reflow Rates and Odds Ratios Across CONUT Score Categories

CONUT Category	n	No-Reflow, n (%)	OR (95% CI) vs Normal†
0–1 (Normal nutritional status)	163	10 (6.1%)	Reference
2–4 (Mild malnutrition)	111	32 (28.8%)	**OR 6.197 (2.898–13.255) p < 0.001**
5–8 (Moderate malnutrition)	5	4 (80.0%)	**OR 22.095 (2.242–217.8) p = 0.003‡**
**Binary: CONUT ≥ 2 vs 0–1**	**116 vs 163**	**36 (31.0%) vs 10 (6.1%)**	**OR 6.885 (95% CI 3.249–14.590) p < 0.001**

† Odds ratios estimated by binary logistic regression (categorical CONUT as predictor); reference category = CONUT 0–1 (normal nutritional status).

‡ Fisher's exact test applied for the moderate malnutrition group (n = 5) owing to small cell size. The wide confidence interval for this group reflects limited precision and should be interpreted with caution.

Binary comparison (CONUT ≥ 2 vs 0–1) uses χ^2 test. No patients had severe malnutrition (CONUT 9–12).

CONUT, Controlling Nutritional Status score; CI, confidence interval; OR, odds ratio.
